# Fabrication of phosphor in glass using waste glass for automotive lighting application

**DOI:** 10.1038/s41598-023-27685-2

**Published:** 2023-03-17

**Authors:** Seung Hee Choi, Seok Bin Kwon, Jung Hyeon Yoo, MinYoung Na, Bo Young Kim, HoShin Yoon, Seoung Hyok Park, Isabel Kinski, Bong Kyun Kang, Dae Ho Yoon, Young Hyun Song

**Affiliations:** 1 Lighting Materials & Components Research Center, Korea Photonics Technology Institute, Gwangju, 61007 Republic of Korea; 2 School of Advanced Materials Science and Engineering, SungKyunKwan University, Suwon, 16419 Republic of Korea; 3 Force4, Gwangju, 61009 Republic of Korea; 4 Department of Electronic Materials and Devices Engineering, Soonchunhyang University, 22, Soonchunhyang-ro, Asan, Chungnam 31538 Republic of Korea; 5 Department of Display Materials Engineering, Soonchunhyang University, 22, Soonchunhyang-ro, Asan, Chungnam 31538 Republic of Korea; 6 Fraunhofer Research Institution for Materials Recycling and Resource Strategies IWKS, Brentanostrasse 2a, Alzenau, 63755 Hermsdorf, Germany

**Keywords:** Environmental impact, Materials for optics

## Abstract

With advancement of technology, requirements for light-emitting devices are increasing. Various types of packaging technologies have been suggested to improve the performance of light-emitting diode (LED). Among them, phosphor in glass (PiG) is attracting attention due to its manufactural facility and easily tunable characteristics. As PiG draws increasing attention, research on glass materials is also being actively conducted. However, studies about glass in the field of phosphor are mainly conducted on fabrication. Only a few studies about recycling have been reported. Thus, the objective of this study was to recycle waste glass discarded in other fields due to breakage and failure and use it to fabricate phosphor in glass. Cylindrical waste glass was pulverized into powder with an average size of 12 μm, mixed with a phosphor and sintered to be reborn as a phosphor in glass to broaden the recycling route for waste glass.

## Introduction

In the age of Net-Zero, white light-emitting diodes (WLEDs) have received great attention in the global high-power LED market^1–3^. They are mainly used in technologies that output high bright light in fields of backlight, plant growth and automotive lighting^4–6^. WLEDs currently used commercially are implementing a technology using a complementary color relationship by combining a blue InGaN blue chip with a yellow cerium doped yttrium aluminate garnet (YAG: Ce^3+^) phosphor mixed with silicon resin. However, silicone resin generally used for packaging YAG:Ce^3+^ phosphor is not suitable for high-power LED applications because those polymers can be easily damaged by heat or start to darken with time and UV irradiation^1^. To replace those, various types of packaging technologies such as single crystal (SC), ceramic phosphor (CP) and phosphor in glass (PiG) have been developed^7–9^. Among them, SC and CP can lead to better thermal and optical properties than other types. However, their manufacturing process is complicated, requiring very high temperature conditions^10,11^. Therefore, PiG has attracted lots of attention as a technology that can satisfy conditions such as high luminescence performance and low manufacturing cost^12,13^. Furthermore, compared to other types, PiG can be manufactured by combining multiple PiG components with the advantage of easily controlling the emission of PiG^14–16^.

Glass is used in various fields for many kinds of products, such as plate glass, bottle glass, LCD glass and fluorescent lamp glass in everyday life due to its advantages such as excellent optical properties, chemical stability, and low cost^17–19^. Glass recycling is very important in terms of resource saving, energy saving and waste disposal. Waste recycling limits the negative environmental impact of human industrial activities by reducing the production of raw materials^20–22^. In general, waste glass is recycled by sorting the type according to the purpose. However, most recycled waste glass has no record of use except for bottle making and construction aggregates^23,24^. It is necessary to continuously develop technologies to use waste glass, which is difficult to recycle due to breakage, as a raw material for other applications it would have to be high temperature process using a lot of energy^25^.

In this study, we report the fabrication of PiGs using waste glass that cannot be used due to breakage and defects. Waste glass, which was initially in the form of a cylinder, was prepared in a powder form through a coarse grinding/fine grinding process. It was then mixed with a phosphor to prepare PiGs. Results showed that PiGs manufactured from waste glass could achieve superior quality to commercial products. Yellow and amber PiGs manufactured using waste glass carried out in this study can be applied to headlights and side turn signals while complying with carbon reduction policies and reducing resource waste.

## Experimental section

### Preparation of fine glass frit

Cylinder-shaped waste glass was hammered into small chunks. The shattered glass was coarsely ground in a mortar into smaller particles, which were then finely ground by putting them together with a ZrO_2_ ceramic ball in a planetary mixer. A glass frit was obtained by finally filtering the pulverized sample through a sieve.

### Fabrication of yellow and amber PiGs

PiGs were prepared by mixing the glass frit mentioned in the previous section and yellow YAG: Ce^3+^ and amber europium-doped Ca_n-1.5x_Si_12-m-n_Al_m+n_O_n_N^16-n^ (Ca-α-SiAlON) phosphors, respectively. Glass frit, phosphor, Ethanol, and mixing ball were put in a mixing bottle and sealed with parafilm. The cover was closed and milling was performed. The content ratio of yellow or orange phosphor and the glass frit was 1:5. After mixing, samples were quantified and pellets were prepared in a size of 3 inches. The prepared pellets were heat-treated at 650 °C for 1 h in an air atmosphere. They were then polished and diced to prepare square-shaped phosphor converters.

**Figure 1:**
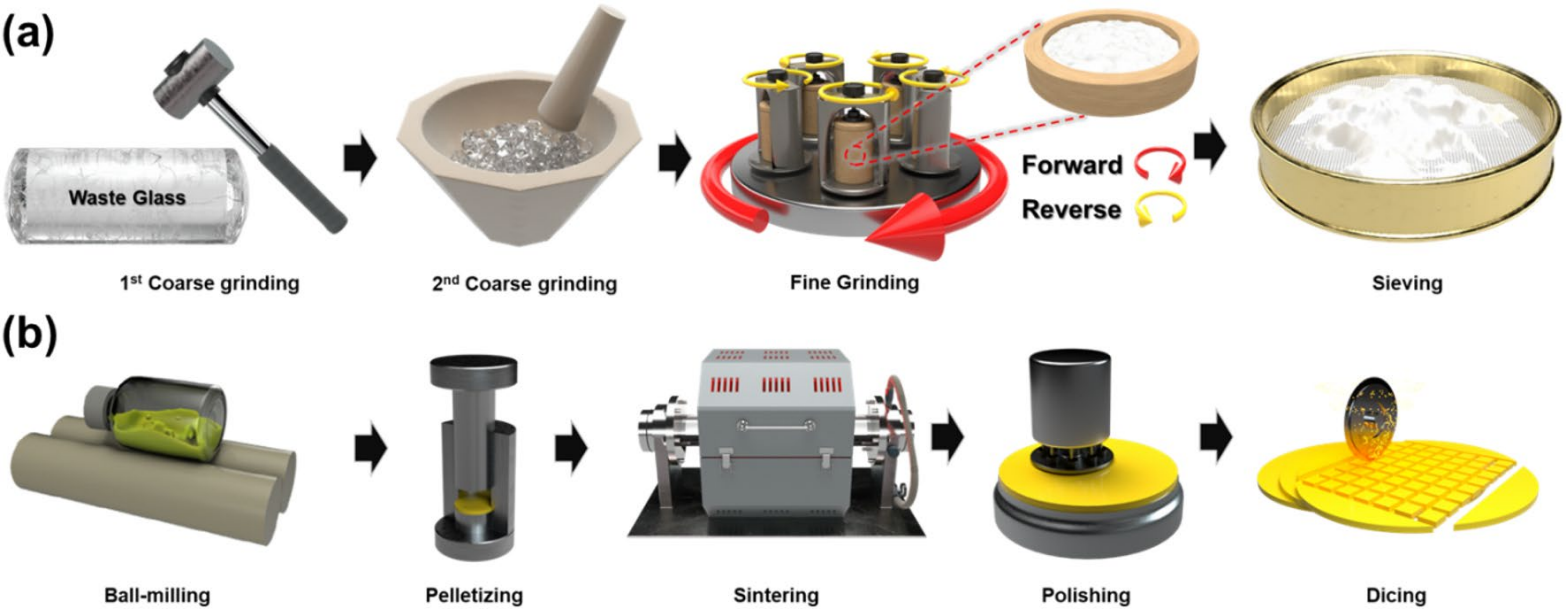
(**a**) Pulverizing process of cylindrical waste glass. (**b**) Fabrication process of PiG.

A detailed process for preparing glass frits and PiGs is shown in Fig. 1.

### Characterization of PiGs

X-ray diffraction (XRD, Bruker D8-Advance) analysis was performed to evaluate the crystallinity of synthesized materials. Optical characterization of PiGs was performed by fluorescence spectroscopy (Fluorescence, Scinco, FS-2, Korea). Electroluminescence characteristic was performed by integrating spheres (PSI Co., Ltd/Korea) under blue LED. Particle morphology and chemical composition were measured by field emission scanning electron microscopy (FE-SEM, JEOL, JSM-7600F with Energy Dispersive Spectroscopy (EDS)). For the surface morphology and component analysis of PiGs, the specimens were processed with an ion-beam cross-section polisher.

## Results and discussion

**Figure 2:**
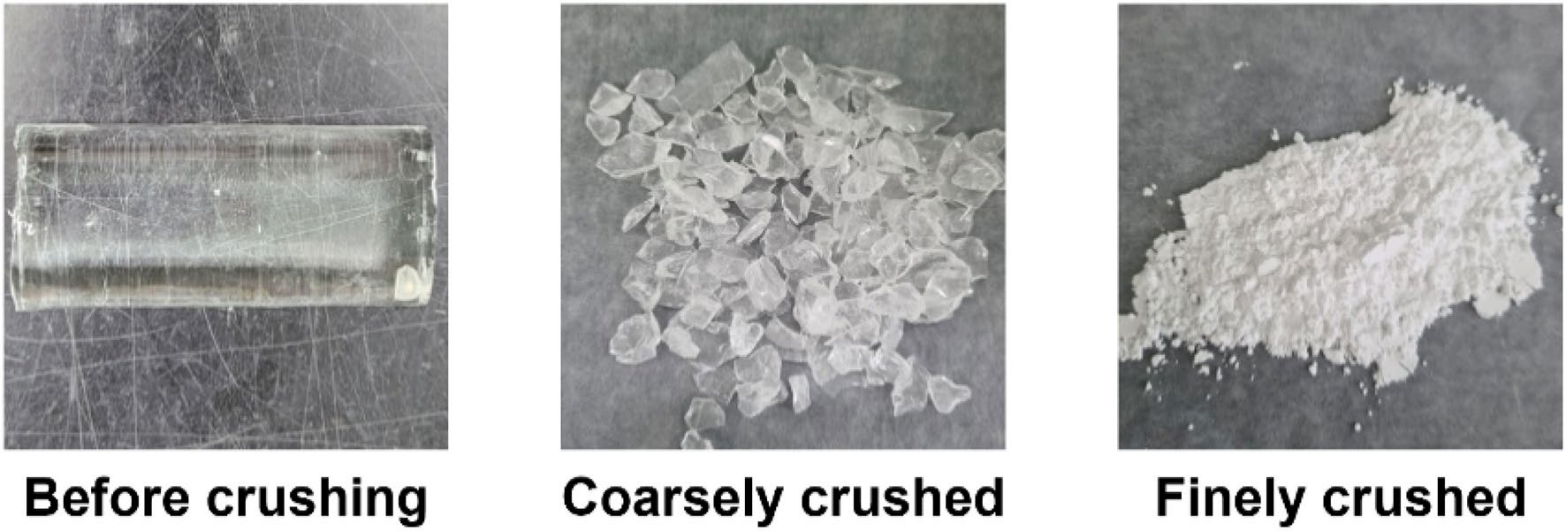
Photographs of glass for each step.

Cylindrical waste glass that could not be used for their original purposes due to fracture, failure, or scratches were ground in coarse and fine processes to fabricate glass frit. The prepared glass frit from the waste glass was applied to make PiGs by combining it with phosphor. Figure 2 shows morphology changes of waste glass in the grinding process.

To confirm the reproducibility and yield of the grinding process, six specimens fabricated under the same condition were compared. When 300 g of waste glass was put in the grinding process under 500 rpm for 20 min, 185 g of glass frits was obtained with an average yield of 62%. Exact values are displayed in Table 1.

**Table 1:** Working conditions of fine grinding process (specimens 1 to 6).

20 mm ceramic ball (ea)	Glass (g)	RPM	Min	Sieve (53 μm)	Yield (g)	Yield (%)
30	300	500	20	O	191	63.67
30	300	500	20	O	185	61.67
30	300	500	20	O	190	63.33
30	300	500	20	O	191	63.67
30	300	500	20	O	189	63
30	300	500	20	O	187	62.33

**Figure 3:**
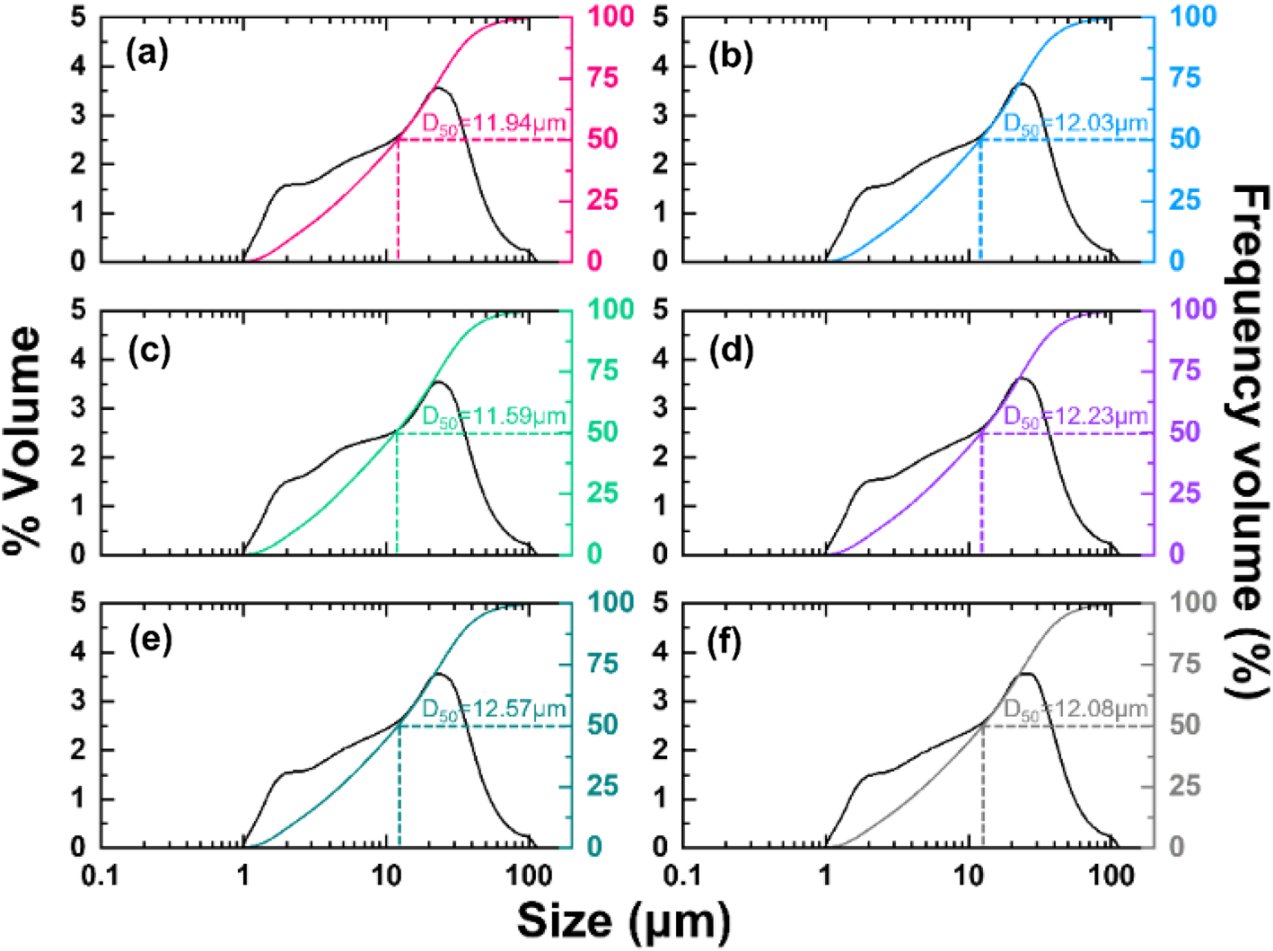
PSA results of glass frit under the same condition. (**a**–**f**) Specimens 1 to 6.

To investigate particle size distribution trend of fabricated glass frit, particle size analysis (PSA) was performed. Figure 3a–f show a similar graph trend and mean particle volume at D (10), D (50) and D (90). Average volumes are exhibited as 2.241 μm for D (10), 12.07 μm for D (50) and 36.275 µm for D (90). Those values had a maximum error of 2.46%, 4.08% and 3.71%, respectively. Such PSA result implies that the glass frit fabrication process for recycling the waste glass has reliability to be applied in a practical application.

**Figure 4:**
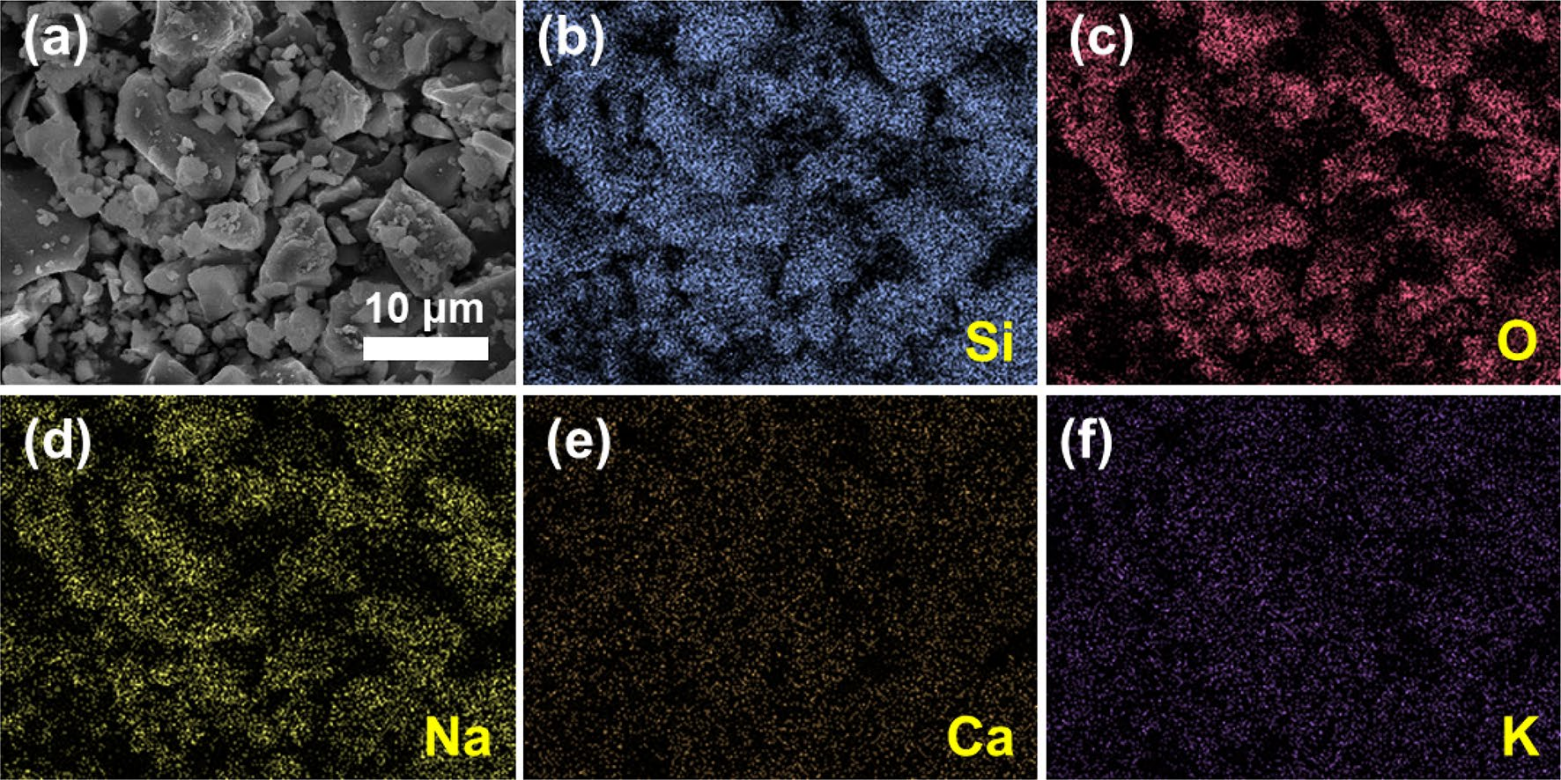
(**a**) SEM images of fabricated glass frit. (**b**–**f**) EDS mapping images.

SEM image of glass frit in Fig. 4a visually shows a result consistent with PSA results. Particles with a size of around 12 μm similar to size of D (50) mainly occupied the volume, with many smaller particles occupying the remaining volume. To figure out the exact composition of waste glass, EDS analysis was performed, as shown in Fig. 4b–f. Results revealed that Si, Ca, Na, O, K were detected as main components. Those are usually used in soda-lime glass which is regarded as a re-usable glass. The soda-lime glass contains both sodium and calcium and is well known as the most commonly used glass in our daily life. Such a glass material may vary significantly in glass transition temperature and transmittance according to sodium oxide and calcium oxide content. Therefore, additional analyzes were required to establish the PiG manufacturing process and prove that it is a suitable material.

**Figure 5:**
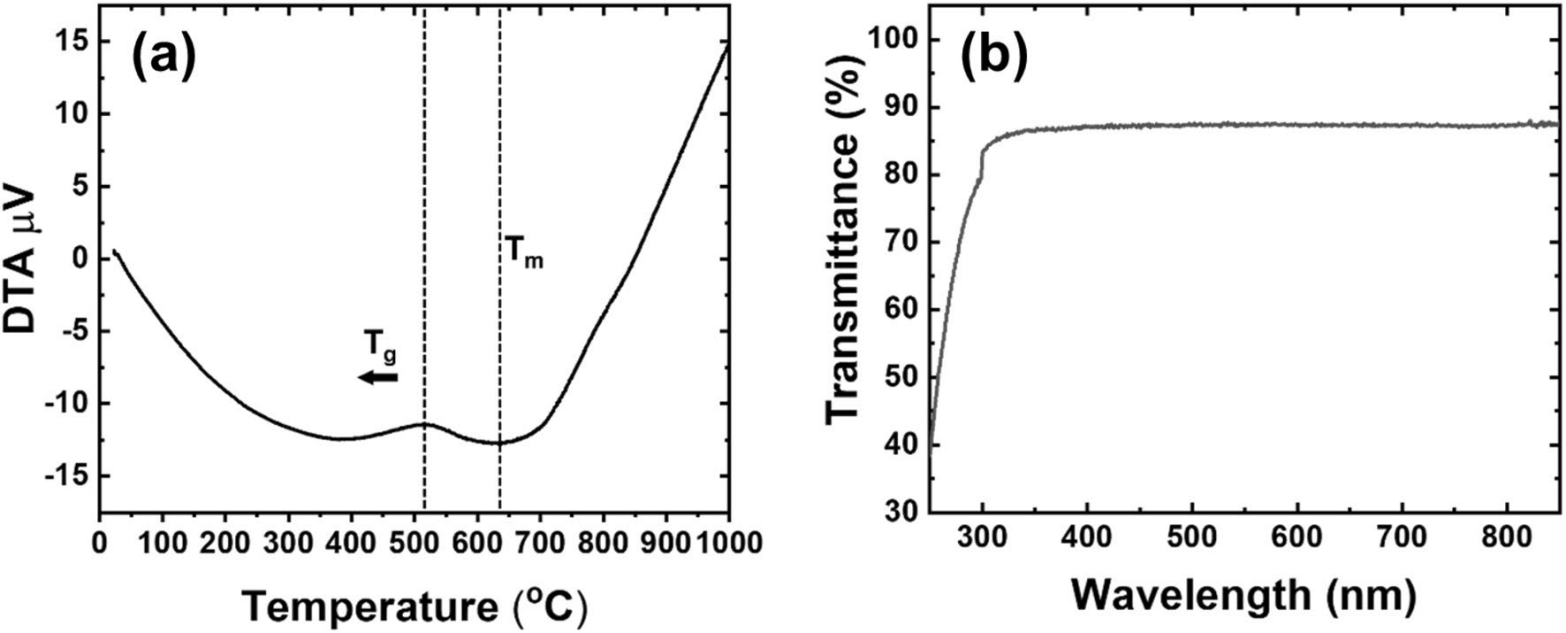
(**a**) DSC analysis result of glass frit. (**b**) Transmittance of fabricated glass disk.

In order to find further characteristic and sintering temperature, Differential Thermal Analysis (DTA) was performed. As shown in Fig. 5a, an obvious endo-thermal at a temperature of 630 °C was observed. This sole peak existed for the whole temperature range. This result implies that the temperature at this peak is melting temperature (T_m_). Based on the analysis, the sintering temperature was determined, and PiGs were fabricated. Figure 5b shows results of transmittance analysis of the glass disk fabricated by melting the prepared glass frit. As shown in the graph, the glass disk performed an excellent transmittance of over 85% for the whole wavelength above UV region of 320 nm. This result also supports the possibility of glass recycling and application for PiG fabrication.

**Figure 6:**
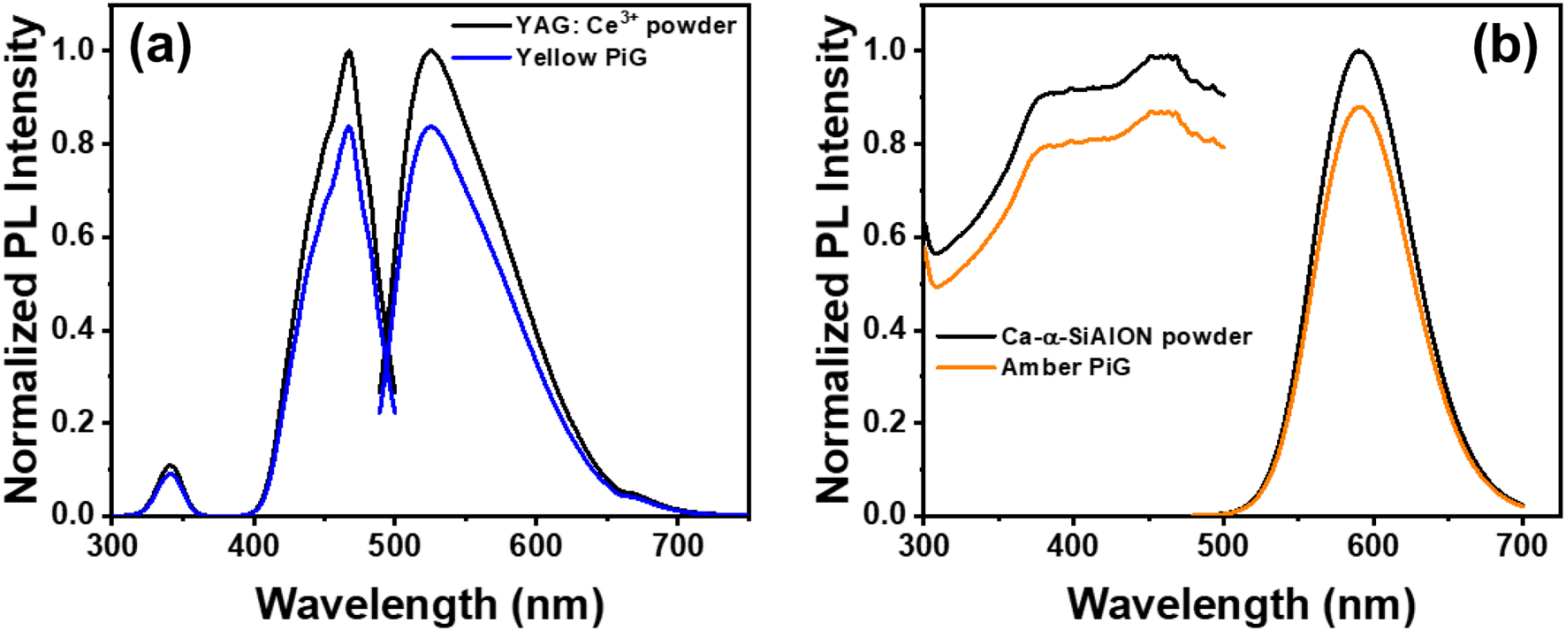
PL analysis of (**a**) YAG: Ce^3+^ powder and yellow PiG and (**b**) Ca-α-SiAlON powder and amber PiG.

To manufacture a PiG using waste glass, we selected YAG: Ce^3+^ as a yellow phosphor and Ca-α-SiAlON as an amber phosphor. Figure 6 shows the results of evaluating the photoluminescence characteristics of the two selected phosphors and PiGs prepared using these phosphors. As shown in Fig. 6a,b, both phosphors excited with a wavelength of 450 nm in the blue region (see excitation spectra left curves), and each phosphor also showed emission bands centered at about 530 nm and 600 nm by the excitation source. These emission spectra are attributed to the 5d^1^–4f transition of Ce^3+^ (see Fig. 6a, right curve) and the 4f^6^5d–4f^7^ transition of Eu^2+^ (Fig. 6b, right curve), respectively^26,27^. In addition, the fact that there is no difference from the unique optical properties of the powder when manufactured with PiG confirms that the phosphor does not react with the glass composition, and the structure does not collapse during the sintering process^28^.

**Figure 7:**
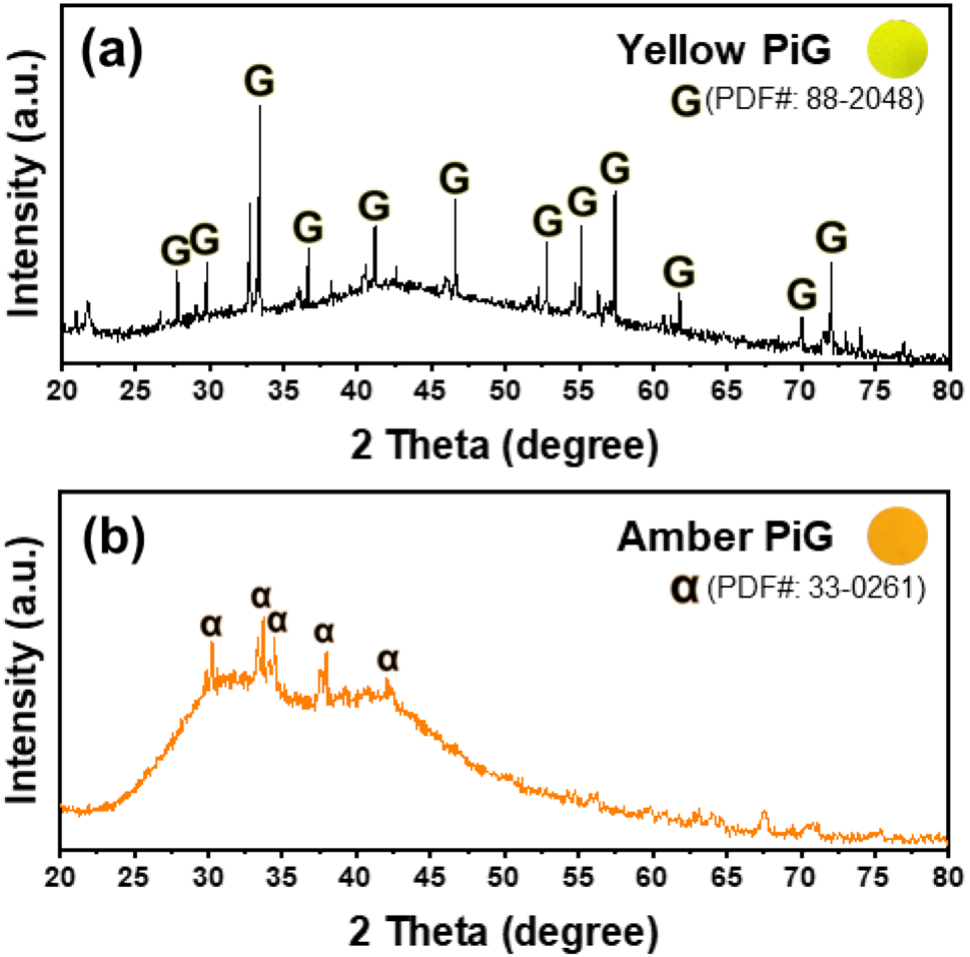
XRD patterns of fabricated yellow (**a**) and amber (**b**) PiGs.

**Figure 8:**
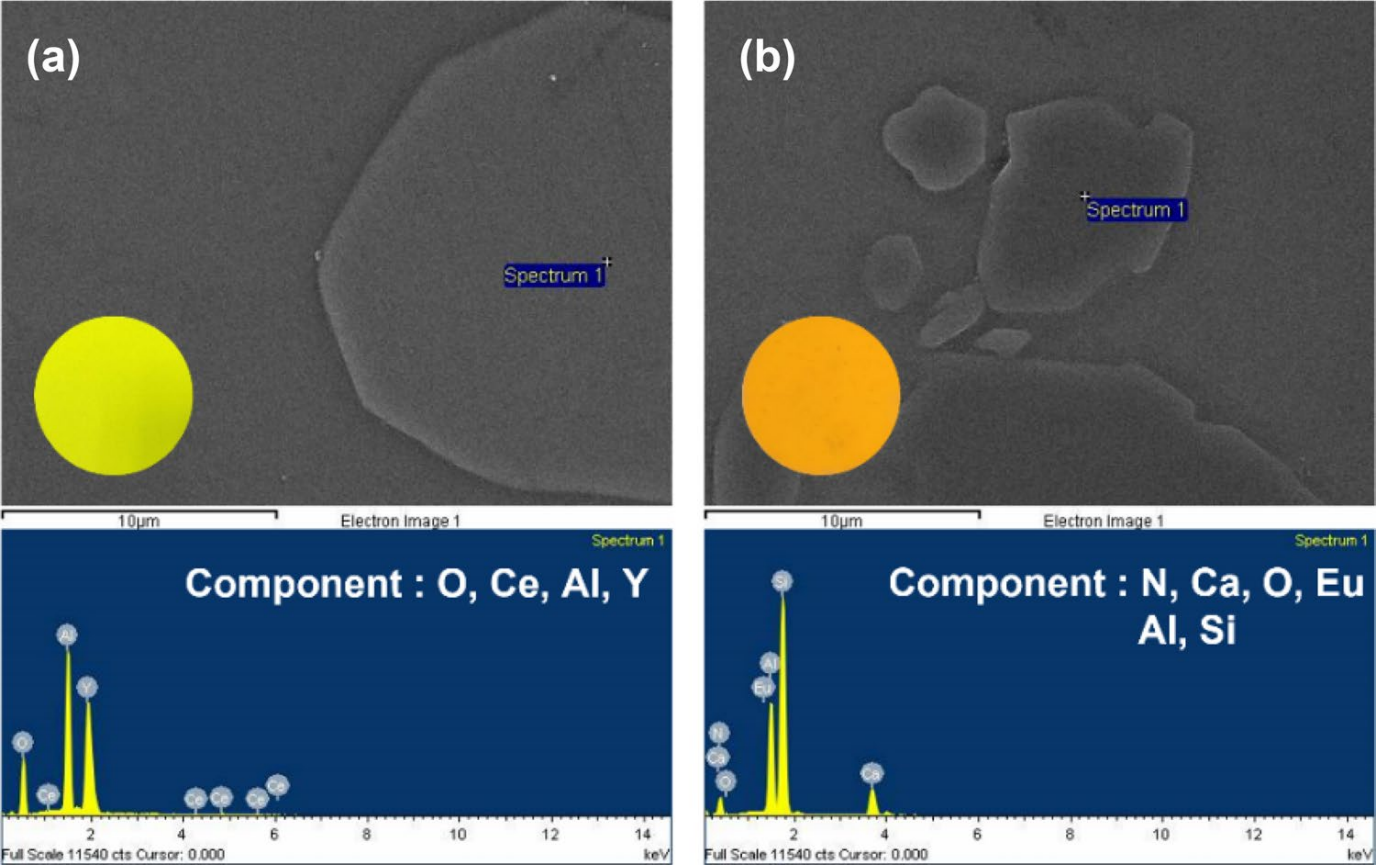
Point EDS results of yellow and amber PiGs, respectively.

PiGs were manufactured using waste glass with selected phosphors. XRD pattern analysis was performed to evaluate whether the structure of the phosphor was destroyed due to internal reaction during the manufacturing process. In XRD results shown in Fig. 7, the broad diffraction for an amorphous glass of the soda-lime glass was detected in the range of 25°–50° for the two PiGs, and XRD powder diffraction patterns of phosphors were clearly revealed. This result suggested that the phosphor maintains the structure well without collapsing inside the glass. In addition, the results of point EDS analysis to confirm that the phosphor is well maintained inside the glass material are shown in Fig. 8a,b. As a result of analyzing the composition of the portion presumed to be the phosphor particles, each phosphor component was accurately detected.

**Figure 9:**
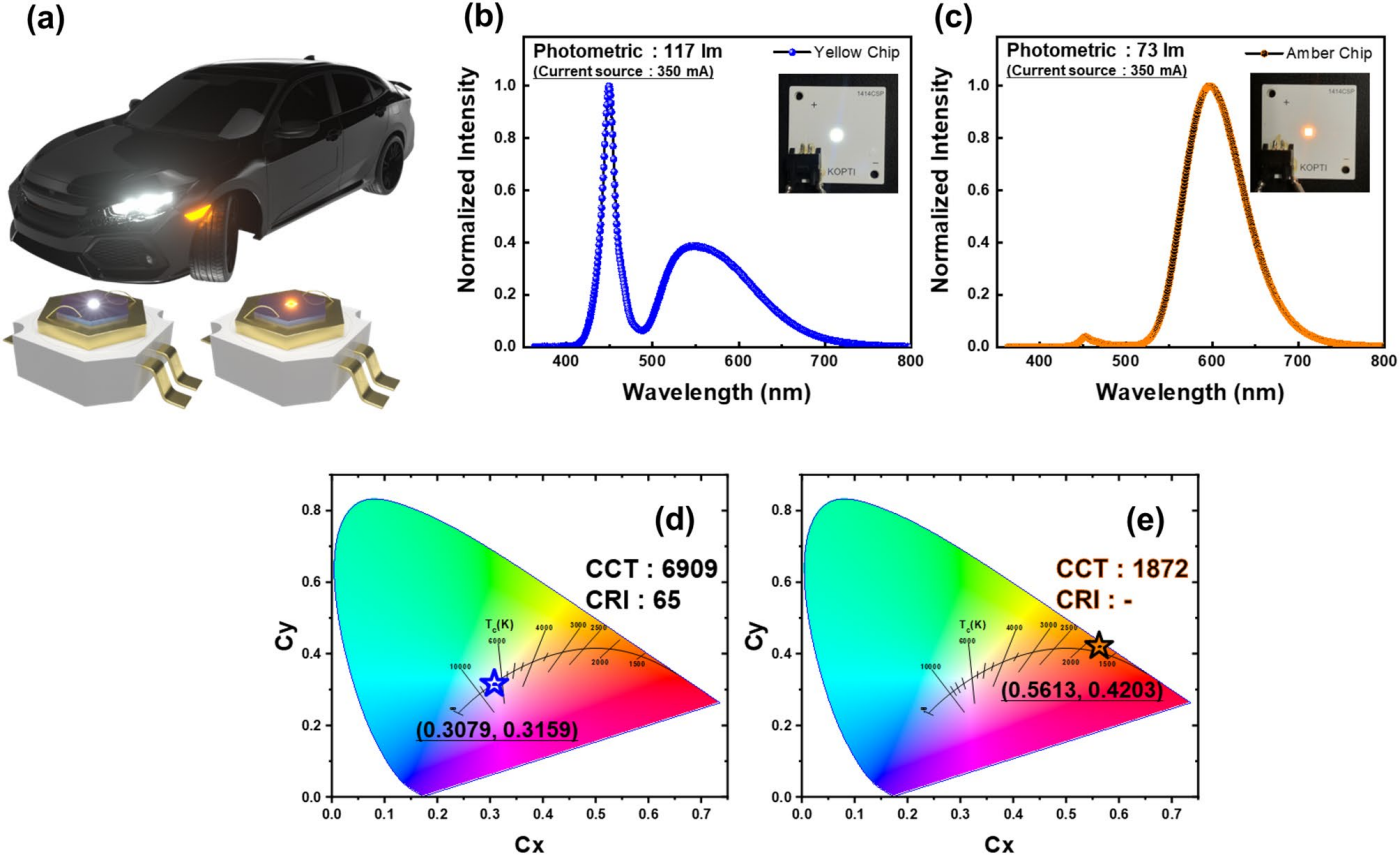
(**a**) Application example of fabricated PiGs (**b**), (**d**) Package test result of Yellow PiG (**c**), (**e**) Package test result of Amber PiG.

In order to analyze optical characteristics of PiGs successfully manufactured using waste glass, a blue-chip package test was conducted. Figure 9a shows a schematic diagram of automotive lighting applications that can be applied when modularized with a blue LED-based light converter. Figure 9b,c show results of optical properties measured in the integrating sphere by packaging PiGs manufactured in size of 1.375 mm^2^ with a blue LED chip. Yellow and amber PiGs had luminous flux values of 117 lm and 73 lm under 350 mA driving current, respectively. As shown in Fig. 9d,e, the Blue-Yellow LED device realized color coordinates that matched white LED standards and the Blue-Amber LED device realized color coordinates that could be applied to side turn lamps.

## Conclusion

In summary, to broaden the recycling field of waste glass to reduce raw material consumption, we pulverized waste glass to fabricate PiG. Waste glass, which was difficult to be used for its original purpose due to scratches and cracks in the produced glass, was pulverized to an average particle size of 12 µm through coarse grinding and fine grinding. Based on the glass transition temperature obtained through DSC analysis, sintering conditions of PiGs were optimized and yellow and amber PiGs were successfully prepared. Under blue LED excitation, yellow PiG showed luminous flux of 117 lm and (0.3079, 0.3159) color coordinates and amber PiG showed luminous flux of 73 lm and (0.5613, 0.4203) color coordinates. We demonstrated the excellent value of waste glass utilization in the field of remote phosphors, and we believe that this will be a stepping stone for overcoming limitations in the field of recycling.

## Data Availability

The data that support the findings of this study are available within the article.
