# Author Correction: Fabrication of phosphor in glass using waste glass for automotive lighting application

**DOI:** 10.1038/s41598-023-34180-1

**Published:** 2023-05-09

**Authors:** Seung Hee Choi, Seok Bin Kwon, Jung Hyeon Yoo, MinYoung Na, Bo Young Kim, HoShin Yoon, Seoung Hyok Park, Isabel Kinski, Bong Kyun Kang, Dae Ho Yoon, Young Hyun Song

**Affiliations:** 1 Lighting Materials & Components Research Center, Korea Photonics Technology Institute, Gwangju, 61007 Republic of Korea; 2 School of Advanced Materials Science and Engineering, SungKyunKwan University, Suwon, 16419 Republic of Korea; 3 Force4, Gwangju, 61009 Republic of Korea; 4 Department of Electronic Materials and Devices Engineering, Soonchunhyang University, 22, Soonchunhyang-ro, Asan, Chungnam 31538 Republic of Korea; 5 Department of Display Materials Engineering, Soonchunhyang University, 22, Soonchunhyang-ro, Asan, Chungnam 31538 Republic of Korea; 6 Fraunhofer Research Institution for Materials Recycling and Resource Strategies IWKS, Brentanostrasse 2a, Alzenau, 63755 Hermsdorf, Germany

Correction to: *Scientific Reports* 10.1038/s41598-023-27685-2, published online 17 March 2023

In the original version of this article Seung Hee Choi, Bo Young Kim and Young Hyun Song were incorrectly affiliated with ‘Mobility Lighting Research Center, Korea Photonics Technology Institute, Gwangju, 61007, Republic of Korea’. The correct affiliation is listed below.

Lighting Materials & Components Research Center, Korea Photonics Technology Institute, Gwangju 61007, Republic of Korea.

The original Article has been corrected.

